# Synthesis of scale‐dependent plant biodiversity change in climate warming experiments

**DOI:** 10.1002/ecy.70486

**Published:** 2026-09-22

**Authors:** Lotte Korell, Martin Andrzejak, Jonathan M. Chase, W. Stanley Harpole, Tiffany M. Knight

**Affiliations:** ^1^ Department of Community Ecology Helmholtz Centre for Environmental Research (UFZ) Halle (Saale) Germany; ^2^ Department of Species Interaction Ecology Helmholtz Centre for Environmental Research (UFZ) Leipzig Germany; ^3^ German Centre for Integrative Biodiversity Research (iDiv) Halle‐Jena‐Leipzig Leipzig Germany; ^4^ Ecology and Genetics Unit University of Oulu Oulu Finland; ^5^ Martin Luther University Halle‐Wittenberg Halle (Saale) Germany; ^6^ Department of Physiological Diversity Helmholtz Centre for Environmental Research (UFZ) Leipzig Germany

**Keywords:** beta diversity, biodiversity change, climate change, global warming, spatial scale, terrestrial plant communities, warming experiments

## Abstract

Climate warming is one of the most pressing environmental challenges for plant populations, communities, and ecosystems on earth. Warming experiments are an important tool for establishing causal relationships between climate change and plant diversity responses. However, results from such experiments vary in direction and magnitude. We hypothesize that such variation arises from context‐dependent factors, such as the magnitude of the temperature manipulation, the study duration, the spatial grain of the analysis, the background climate in which the study was conducted, and the biodiversity metrics used. Here, we present a comprehensive synthesis of primary data from 42 warming experiments, mostly from tundra and grassland ecosystems. We examined how warming influenced composition and how responses of plant diversity varied at the alpha (= plot), beta (= plot‐to‐plot), and gamma (= site) scales. Context‐dependent factors explained some of the variation observed in responses across studies. Higher magnitudes of warming led to stronger declines in species richness at the alpha scale. Experimental warming led to stronger declines in species richness in plant communities with warmer background summer temperature (e.g., desert grasslands) compared to those in communities exposed to colder background summer temperatures (e.g., tundra communities). Warming decreased evenness at the local (alpha) scale, but increased evenness at the beta scale, indicating that replicate warming plots diverged in their abundance distribution over time. Warming changed the composition of plant communities, particularly through species turnover rather than in the nestedness component, suggesting that warming leads to novel species assemblages. Our study highlights that responses of terrestrial plant communities to warming depend on experimental methodology, environmental context, and the spatial grain of investigation.

## INTRODUCTION

Climate warming is a key driver of global environmental change (IPCC, 2022). Without drastic reductions in greenhouse gas emissions, climate warming will likely exceed 2°C by 2100 (IPCC, 2022; Raftery et al., 2017). Climate warming has various effects on plant communities that manifest at different spatial and temporal scales. At the alpha scale (i.e., the local or plot scale), species with limited capabilities to adapt to climate warming will face local extinctions due to rising temperatures, reducing the species richness of the community (Panetta et al., 2018; Thomas et al., 2004; Urban, 2024). However, the extinction process is often slow for terrestrial plants, due to long life spans of perennial species and the time it takes to deplete soil seed banks (Bertrand et al., 2016; Richard et al., 2021; Zhu et al., 2024). Local changes in composition can be even stronger than changes in diversity (Bastazini et al., 2021; Harrison, 2020). For example, warming in colder systems has been shown to increase the dominance of plant species with rapid growth potential and tall stature, altering community composition more significantly than diversity (Harrison, 2020). At larger spatiotemporal scales (e.g., regional or continental scales), plant species have been shown to shift their ranges towards the poles and higher altitudes as a consequence of climate warming (see meta‐analysis by Parmesan & Yohe, 2003).

Much of our knowledge on the effects of global change, including climate warming, on plant communities stems from long‐term observational studies (e.g., Antão et al., 2020; Feeley et al., 2020; Jandt et al., 2022; Midolo et al., 2025; Steinbauer et al., 2018; Vellend et al., 2013) and species distribution models (e.g., Thomas et al., 2004; Thuiller et al., 2005). Such observational studies are highly valuable; however, it remains difficult to disentangle the effects of warming from other co‐varying factors. Climate change experiments that manipulate temperature and measure plant community responses provide a powerful tool to establish causal driver‐response relationships (Thompson et al., 2013), and the number of these studies has been steadily increasing over the last 20–30 years (Gruner et al., 2017; Korell et al., 2020; Song et al., 2019). The results of these studies vary both in the magnitude and direction by which warming influences the structure and diversity of plant communities (e.g., Gough & Hobbie, 2003; Hollister et al., 2005; Reed et al., 2021). This is likely a result of numerous context‐dependent factors, and thus the growth in the number of experimental results available enables this new synthesis research to disentangle the factors that moderate the effects of climate warming on terrestrial plant communities.

Context‐dependent factors that might moderate plant community responses to experimental warming include: the magnitude of temperature increase, background temperature conditions in which the study was conducted, the study duration, the spatial grain of the analysis, and the biodiversity metrics analyzed (Elmendorf et al., 2012; Komatsu et al., 2019; Korell et al., 2021). Depending on the emissions scenario and geographical region, global circulation models project temperatures likely rising 2–4°C by 2100 (IPCC, 2022). Warming experiments thus vary considerably in the magnitude of their manipulated temperature increase (Korell et al., 2020; Song et al., 2019), and more intense manipulations can lead to larger plant community responses (Korell et al., 2021). Likewise, background temperature conditions during the growing season (i.e., summer temperatures) can influence plant community responses to temperature increases (Elmendorf et al., 2012; García Criado et al., 2025). Locations that are already warm are frequently also water limited (e.g., arid ecosystems), and experimental warming, especially during the growing season, could exacerbate this water limitation for plants, causing more dramatic decreases in species diversity with rising temperatures (De Boeck et al., 2016; Harrison, 2020; Lewandrowski et al., 2021). In contrast, for communities with colder background temperature conditions, warming could have milder or even positive effects on biodiversity, for example by increasing the length of the growing season and allowing colonizing species from warmer regions to establish (García Criado et al., 2025; Harrison, 2020; Midolo et al., 2025; Steinbauer et al., 2018). However, such reshuffling of plant communities with rising temperatures could also occur at the expense of more specialized species adapted to harsher climate conditions, that are often less competitive under more benign conditions (Markley et al., 2025). Plants take time to demographically respond (mortality, growth, recruitment) to experimental changes in temperature, and thus effects of warming on diversity should increase with the duration of the experiment. This might be particularly relevant in communities with cold background temperature conditions composed of slow‐growing perennial plant species (Elmendorf et al., 2012; Hollister et al., 2005).

The metrics by which diversity and composition are measured, as well as the spatial scale on which observations are made can also affect the responses of plant diversity to warming (Avolio et al., 2021; Chase et al., 2018; Hillebrand et al., 2008; Spake et al., 2021). If warming decreases the total abundance of plants and/or decreases the evenness of the plant community, then species richness would decrease at the alpha scale (Chase et al., 2018; He & Legendre, 2002; Srivastava & Lawton, 1998), but not at larger scales (e.g., at the gamma = site scale) so long as at least one individual of the species is found across all replicates of a treatment. Decreases in evenness might occur if, for example, climate warming increases the relative abundance of the species that can tolerate warmer and drier conditions, such as deep‐rooted grasses and forbs (Liu et al., 2018). Alternatively, if warming treatments homogenize plant communities (i.e., decrease Whittaker's beta diversity), then decreases in species richness will be stronger at larger spatial grains compared to smaller spatial grains (Vellend et al., 2017). This might occur if, for example, climate warming creates a strong environmental filter that reduces the role that environmental heterogeneity across plots can have on beta diversity (Stewart et al., 2018).

Climate warming can influence plant diversity through changes in the number of species, as well as through changes in the identity of species, that is, compositional changes between warmed and control plots. Whereas Whittaker's beta diversity refers to changes between local and regional scales (Whittaker, 1972), dissimilarity indices refer to pair‐wise changes in the identity of species between two types of communities, such as, treated versus control communities. This differences in species composition due to warming can be more effectively understood by decomposing total dissimilarity into two components: turnover (species replacement between warming and control treatments) and nestedness (where the climate warming treatment has a subset of the species present in the control treatment, Baselga, 2017). Nestedness signals non‐random patterns of species loss or gain with warming, reflecting environmental filtering (Baselga, 2017; Baselga et al., 2007). In contrast, turnover indicates that warming creates novel communities that may be harder to predict (Hillebrand et al., 2010; Pinsky et al., 2025; Urban et al., 2012; Williams & Jackson, 2007).

To date, meta‐analyses of terrestrial plant warming experiments have focused on a single spatial grain (typically the plot scale) and/or a single biome (e.g., tundra) and showed that warming tends to decrease local plant biodiversity (Elmendorf et al., 2012; Gruner et al., 2017; Shangguan et al., 2024; Zheng et al., 2026). Another synthesis considering multiple taxa (fungi, animals, plants) and multiple spatial grains (i.e., alpha, beta, gamma) found that warming tended to increase beta diversity (Bastazini et al., 2021). While this study had limited sample sizes for terrestrial plants, and could therefore not test many of the context dependencies described above, it highlights the importance of examining effects of warming on biodiversity at multiple spatial grains.

Here, we provide a comprehensive synthesis on the effects of experimental warming on the diversity of terrestrial plant communities across scales, while accounting for differences in the magnitude and duration of simulated climate warming, as well as the background temperature context of the experimental sites. We acquired primary data from 30 studies on the presence and abundance of plant species from 42 experiments, primarily from grasslands but also tundra and shrublands, and examined how warming influenced multiple metrics, including species richness, evenness, and incidence‐ and abundance‐based composition. Across each biodiversity metric and spatial grain, we examined whether the responses to warming depended on the context (magnitude and duration of simulated climate warming, background summer temperature). This comprehensive analysis will provide information about the spatial grain and metrics of the patterns, as well as clues about the potential processes (e.g., environmental filtering, extinction debt) and the predictability of plant communities’ responses to warming.

## METHODS

### Literature review and data extraction

**FIGURE 1:**
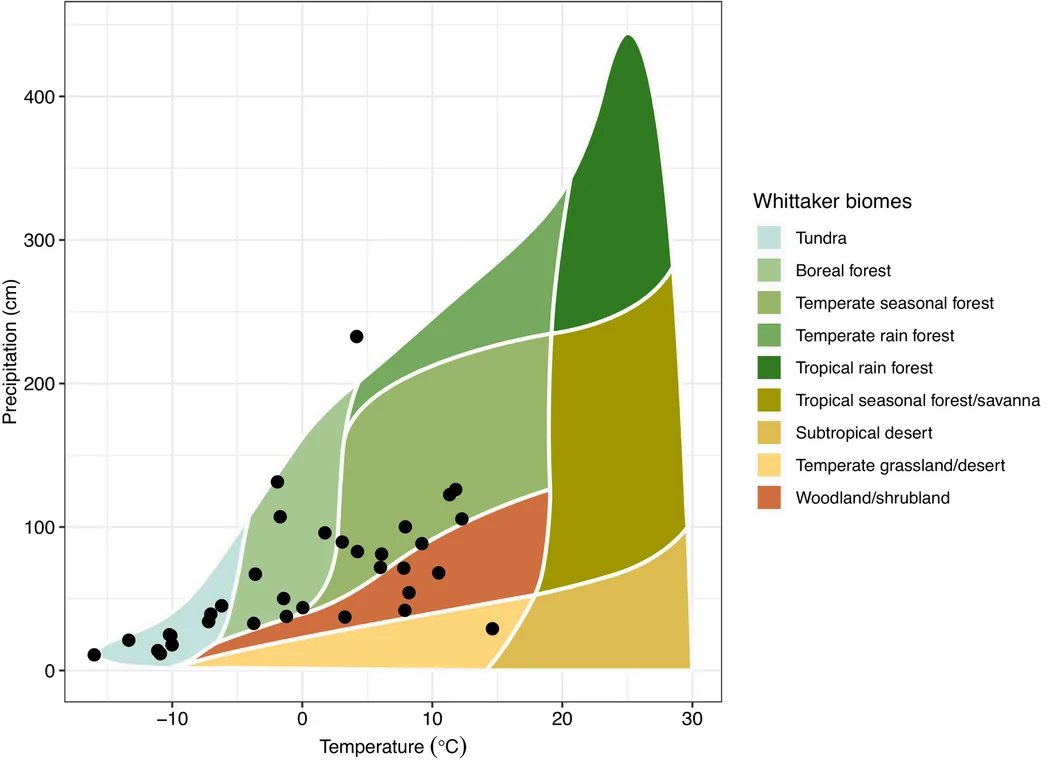
Whittaker's biome plot, defined by mean annual temperature (in centimeters) and mean annual precipitation (in degrees Celsius). Points represent climate manipulation experiments.

We conducted a systematic literature review in ISI web of knowledge and Google Scholar using the search terms (warming OR temp* change OR climate change*) AND (biodiversity OR rich* OR diversity OR abundance OR evenness) AND (plant community OR plant*) AND (terr* ecosystems) AND (field experiment OR climate change experiment OR climate manipulation OR open top chamber OR heat* experiment). We considered primary studies ranging from 1995 until 2023. In all, we found ~1600 published studies from which we screened titles and abstracts (Appendix S1: Figure S1). We included studies if they manipulated temperature in field experiments of terrestrial ecosystems and provided estimates of all vascular plant species present, as well as their relative abundances (or made it clear that such information was available, in which case we contacted authors to request this information). We did not restrict our search to herbaceous systems and our dataset also includes experiments from shrubland and tundra (peatland: 1; grassland: 19; tundra: 22). However, as forests are very hard to manipulate, our dataset does not include forest ecosystems. We excluded pot or mesocosm experiments, as well as experiments that experimentally manipulated or controlled biodiversity (i.e., biodiversity‐ecosystem functioning experiments) because those tended to exclude immigration from the regional species pool, known to affect community responses to warming (Gruner et al., 2017). In all, we found 48 primary studies that met our criteria. Nine of the primary studies provided the needed data in data repositories or supplementary materials, and we requested data from the authors of the remaining 39 primary studies, of which nine provided the needed information. We also followed relevant references from these primary studies, systematic reviews and meta‐analysis, which resulted in 21 additional primary studies and 11 additional studies provided the necessary data. Our final dataset included 30 primary studies (Appendix S1: Table S1) covering various biomes (Figure 1). The choice of what we consider a unique experiment in this synthesis was based on the choices made by the authors of the studies. For example, in five studies similar experimental manipulations were performed at different field sites and in four studies in different community types within the same site. All these experimental manipulations were considered as unique experiments (*n* = 42), but nested hierarchically within the study in all subsequent analyses.

From each primary study, we extracted information about the location of the experiment (i.e., latitude and longitude), the duration of the experimental treatment (number of years), and the magnitude of experimental temperature increase (in degrees Celsius) from ambient temperature at the site. Using each site coordinate, we extracted information on temperature of the warmest quarter (i.e., summer temperature; in degrees Celsius) from the CHELSA database (Karger et al., 2017) to describe the background climatic conditions for each study site. When investigators collected information on community responses to climate manipulations across multiple years and different seasons, we only used the endpoint of the time series at peak biomass for our analysis.

### Measurements of biodiversity across scales

To investigate the effects of temperature on different aspects of biodiversity across scales, we considered two spatial scales. We defined the alpha scale at the individual plot scale (median: 0.75 m^2^, range: 0.16–2.5 m^2^) and the gamma scale as the combination of all observations in a given treatment/site combination (median: 5 m^2^, range: 1.28–12.5 m^2^). Because there were differences in the numbers of replicates in different experiments, we rarefied the gamma scale to the minimum number of replicates observed across experiments (*n* = 3) to make more fair comparisons (Chao et al., 2014).

#### Species richness

We calculated the total number of all observed species at each scale. Alpha scale species richness was calculated as the number of species per plot and (rarefied) gamma scale species richness was calculated as the total number of all species observed in a given treatment/site combination. Because species richness does not include any measures of relative abundance, it provides comparatively more weight to rarer species relative to more common species.

#### Evenness (ENS_Pie_)

To incorporate differences in abundance distribution of species into our diversity metrics, we chose a metric of diversity that more heavily weights common species relative to rare ones—we calculated Hurlbert (1971) probability of interspecific encounter (PIE). The PIE, also known as the Gini‐Simpson index, is represented as a higher weighting of common species on the Hill number continuum (Chao et al., 2014; Hill, 1973; Jost, 2006) (*q* = 2) compared to species richness (*q* = 0) and can be converted to an effective number of species (ENS_Pie_), which represents the number of equally abundant species in a community that would be needed to give the value of PIE (Jost, 2006, i.e., when communities are highly uneven, ENS_PIE_ < < species richness). At both the alpha and gamma scales, we standardized the cover to sum to 100% and used the relative cover abundances per species to calculate ENS_Pie_ at each scale. At the alpha scale, we summed cover across all species, and at the gamma scale, we summed cover abundances across all species and plots per treatment/site combination.

#### Whittaker's beta diversity

To quantify beta diversity as the between‐plot variation in both metrics of diversity (species richness and ENS_Pie_), we used Whittaker's (1972) multiplicative partition of the ratio of gamma‐ and alpha scales (beta = gamma/alpha) (Chao et al., 2014; Tuomisto, 2010; Whittaker, 1972). Higher values of beta diversity for species richness indicate higher plot‐to‐plot heterogeneity in both common and rare species, while higher values in ENS_Pie_ at the beta scale indicate a higher spatial aggregation of common species.

#### Compositional changes

To quantify the effect of warming on species composition, we calculated the pairwise dissimilarity using incidence (i.e., Sørensen) and abundance (i.e., Bray–Curtis) measures between corresponding control and warmed plots within the same block and unique experiment. We partitioned dissimilarities into turnover and nestedness components for incidence and their equivalent components for abundance‐based dissimilarity (Baselga, 2010). Dissimilarity was calculated using beta.pair (incidence) and beta.pair.abund (abundance) functions from the betapart R package (Baselga et al., 2018).

#### Calculation of effect size

Except for the compositional changes where we calculated pairwise differences between control and treated plots, we used the log response ratio (LRR) between experimentally treated and control plots (LRR = ln(treatment) – ln(control)) as an effect size (Hedges et al., 1999). We calculated separate LRRs for each of the biodiversity components at each scale only. A negative effect size of species richness at the alpha‐ and gamma scales indicates a decrease in species richness in response at that respective scale, while a positive effect size of species richness indicates an increase in species richness due to the climate treatment at the respective scale. Similarly, a negative effect size of ENS_Pie_ at the alpha‐ and gamma scales indicates a decrease primarily due to changes in common species, while a positive LRR of ENS_Pie_ indicates an increase primarily due to changes in common species in response to the climate treatment. Negative values in the effect size of species richness and ENS_Pie_ at the beta scale indicate less plot‐to‐plot heterogeneity and less spatial aggregation, while positive values in the effect size of species richness and ENS_Pie_ at the beta scale indicate higher plot‐to‐plot heterogeneity and larger spatial aggregation due to the climate treatment.

### Statistical analysis

For all response variables except composition, we used linear mixed effects model ‘lme4’ package (Bates et al., 2018) with a hierarchical error structure to account for the non‐independence of experiments at the different field sites within a primary study, as well as differences in the experimental designs. The interaction of block, site, and study (alpha‐ and beta scale) and the interaction of site and study (gamma scale) were used as random terms in the models. For each measurement (except composition) and scale, we used separate linear mixed models to test how the duration (in years) of temperature manipulation, the magnitude of temperature manipulation (delta T; in degrees Celsius), and the temperature of the warmest quarter (summer temperature, in degrees Celsius) affected the different diversity measures. We tested for the interactive effect of the magnitude of temperature manipulation and the temperature of the warmest quarter (in degrees Celsius) on the effect size of biodiversity measures across the different scales (Appendix S1: Table S2). For each model set, we generated and evaluated the small sample corrected Akaike information criterion (AICc), ΔAICc, and AICc model weights (w 2) using the dredge function within the MuMIn package (Barton, 2019). We extracted effect size estimates and 95% CIs from the simplest model. We consider an effect size as significant if the 95% CI did not overlap zero. For the compositional change we only tested if warming had an effect on the pairwise dissimilarity and no other moderators were included in the analysis as we had no a priori expectation how they would influence compositional turnover. Both nestedness and turnover were modeled using a zero‐inflated beta distribution with log‐link function using the glmmTMB package (Brooks et al., 2017). All analyses were performed in R v 4.2.2 (R Core Team, 2022).

## RESULTS

### Effects on species richness

**FIGURE 2:**
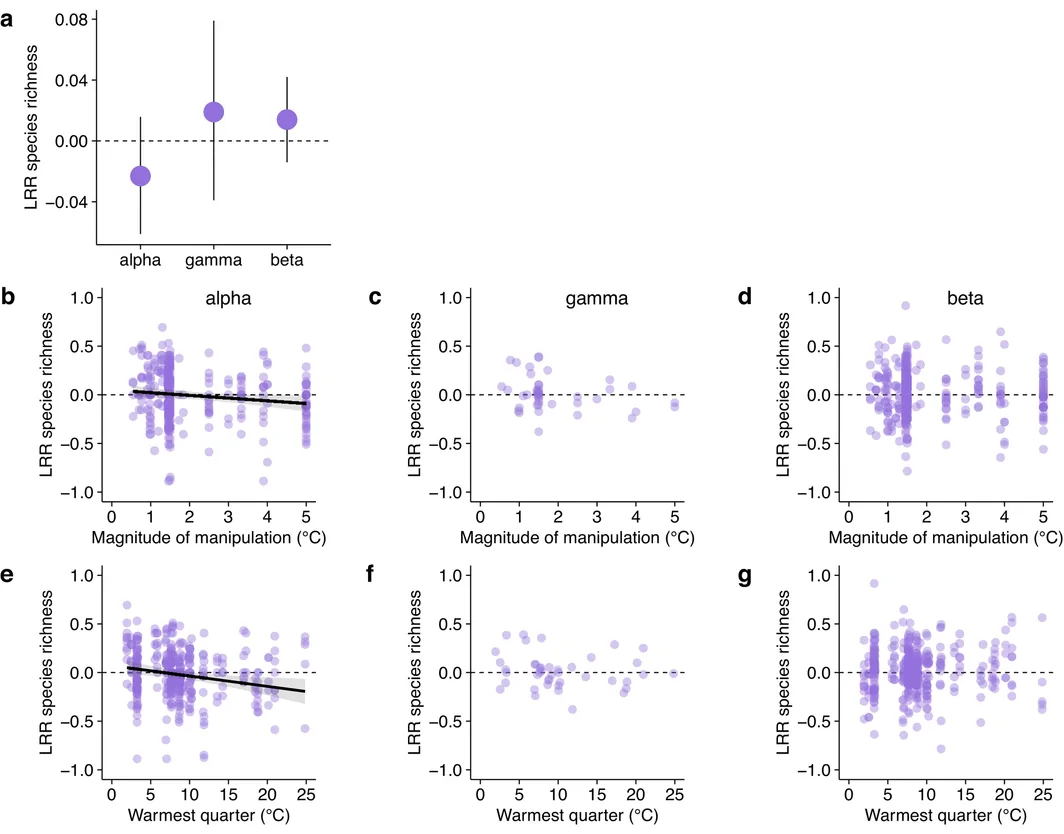
Effects of temperature manipulation on the effect size (LRR = log response ratio) of species richness. Overall effect (grand mean) of climate warming at the alpha, gamma, and beta scales (a); effects of the magnitude of the temperature manipulation (in degrees Celsius) on alpha, gamma, and beta scales (b–d); and effect of background temperature of the warmest quarter (in degrees Celsius) on alpha, gamma, and beta scales (e–g). Data points represent log response ratios of original data (*n* = 344 at the alpha scale, *n* = 42 at the gamma scale). Grand mean and the linear regressions (mean and 95% CI) are based on predicted values of the simplest linear mixed effects model including temperature manipulation (Appendix S1: Table S3).

We detected no significant overall effect of experimental warming on species richness at any scale (Figure 2a). However, we detected a negative effect of warming on species richness at the alpha scale in studies that employed greater levels of experimental warming (mean: 1.9°C, range: 0.75–5°C) and those with warmer background summer temperatures (mean: 10°C, range: 1.9–25°C) (Figure 2b,e). We did not detect effects of these modulating factors on plant species richness responses to warming at the gamma (Figure 2c,f) and beta (Figure 2d,g) scales. We did not find an effect of the experimental study duration (mean: 7 years; range: 1–24 years) on species richness at any scale (Table 1).

**TABLE 1:** Predicted means ±95% upper and lower CI of the linear mixed effects model for the effect size (LRR = log response ratio between treatment and control), and for the different diversity metrics species richness and evenness (ENS_Pie_).

Moderator	Species richness	Evenness (ENS Pie)
Alpha scale		
Grand mean	−0.023 [0.01, −0.06]	**−0.054 [−0.002, −0.10]**
Delta T	**−0.072 [−0.001, −0.13]**	−0.11 [0.06, −0.12]
Duration	−0.09 [0.002, −0.18]	**−0.106 [−0.004, −0.21]**
T warm	**−0.104 [−0.02, −0.17]**	0.009 [0.11, −0.08]
Beta scale		
Grand mean	0.014 [0.04, −0.14]	0.023 [0.07, −0.02]
Delta T	−0.016 [0.04, −0.06]	0.057 [0.15, −0.03]
Duration	−0.013 [0.05, −0.08]	**0.147 [0.26, 0.03]**
T warm	0.029 [0.08, −0.02]	0.095 [0.19, −0.004]
Gamma scale		
Grand mean	0.019 [0.07, −0.03]	−0.025 [0.01, −0.06]
Delta T	−0.086 [0.04, −0.2]	0.037 [0.12, −0.05]
Duration	−0.121 [0.03, −0.26]	0.039 [0.13, −0.06]
T warm	−0.061 [0.03, −0.16]	**0.067 [0.12, 0.009]**

*Note*: Shown is the grand mean and the effect of the different moderators (delta T = magnitude of warming in degrees Celsius, duration = experimental duration (in years), T warm = background summer temperature in degrees Celsius) tested in the analysis. Bold values indicate significant effects.

### Effects on evenness (ENS_Pie_ )

**FIGURE 3:**
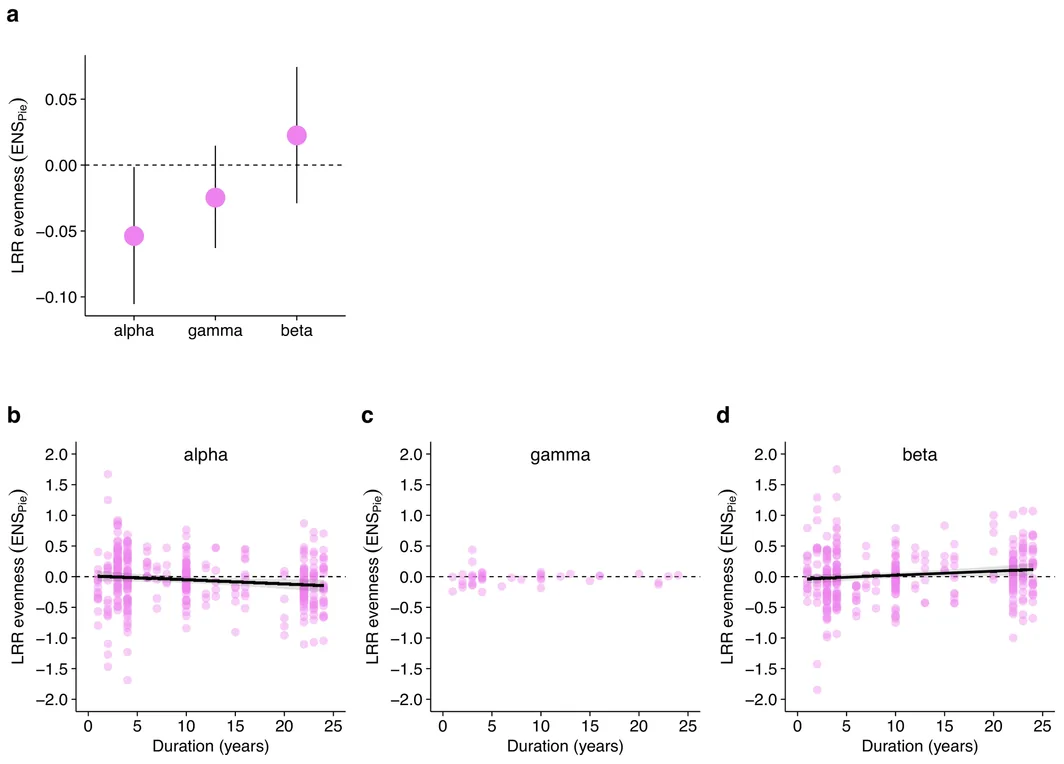
Effects of temperature manipulation on the effect size (LRR = log response ratio) of evenness (ENS_Pie_). Overall effect (grand mean) of climate warming at the alpha, beta, and gamma scales (a); effects of the duration of experimental manipulation (in years) on alpha and gamma (b, c); as well as beta scale (d). Data points represent log response ratios of original data (*n* = 344 at the alpha scale, *n* = 42 at the gamma scale). Grand mean and the linear regressions (mean and 95% CI) are based on predicted values of the simplest linear mixed effects model including temperature manipulation (Appendix S1: Table S3).

Experimental warming had an overall negative effect on evenness (ENS_Pie_) at the alpha scale, but not at the gamma and beta scales (Figure 3a). Furthermore, we detected a negative effect of the study duration on evenness (ENS_Pie_) at the alpha but not the gamma scale (Figure 3b,c), which resulted in a slightly positive effect of study duration on evenness (ENS_Pie_) at the beta scale (Figure 3d). In contrast to species richness, the magnitude of experimental warming and the background climate conditions had no significant effect on the evenness (ENS_Pie_) at any spatial scale (Table 1).

### Effects on composition

**FIGURE 4:**
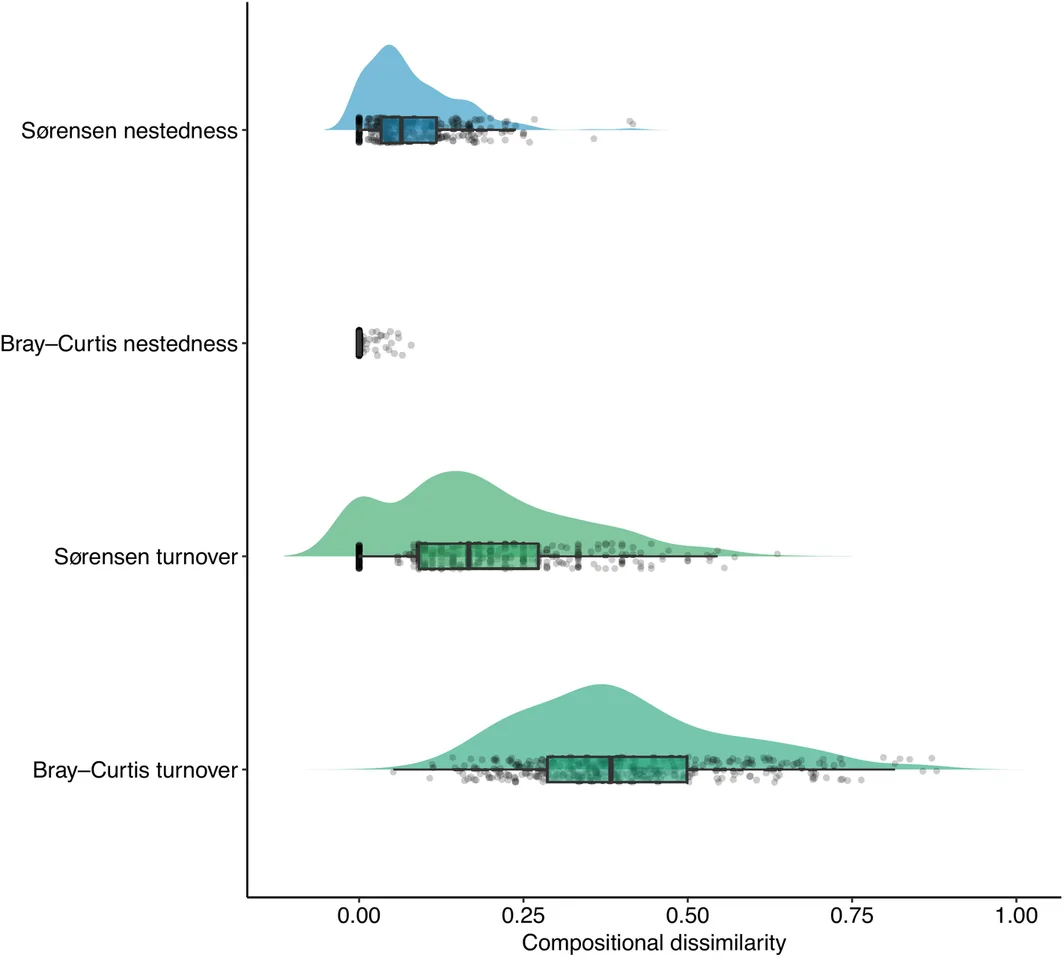
Distribution of compositional dissimilarity values based on Sørensen and Bray–Curtis indices. Each index is partitioned into turnover and nestedness components. Half‐violins represent kernel density estimates, boxplots show the median and interquartile range, and points correspond to individual pairwise comparisons between warmed and control plots.

Experimental warming led to changes in the composition of plant communities (Figure 4). We found that the compositional change measured as the turnover component of dissimilarity was much more apparent than the nestedness component, specifically in the abundance‐based (Bray–Curtis) compared to the incidence‐based metric (Sørensen) (Figure 4).

## DISCUSSION

While climate warming is one of the most severe environmental challenges of this century (IPBES, 2019; IPCC, 2022), its influence on patterns of biodiversity remains nuanced and difficult to predict. Scenario‐based and large‐scale modeling studies suggest that biodiversity will decline at alarming rates with rising temperatures in most regions of the world (Pereira et al., 2024; Thomas et al., 2004). However, syntheses of time series have emphasized that the effects of any environmental driver on plant biodiversity will strongly depend on the local context, and that universal patterns of biodiversity loss in response to climate warming are unlikely (Vellend et al., 2017). For example, colonization by fast‐growing and warmer‐adapted species might curb biodiversity loss for some locations (Harrison, 2020). Long‐term observational studies have thus found both increases and decreases in species diversity with climate warming depending on the location and context (Antão et al., 2020; Jandt et al., 2022; Midolo et al., 2025; Steinbauer et al., 2018; Vellend et al., 2013). Modeling and observational studies struggle to isolate the effects of warming on plant communities amidst the many possible confounding factors, and are limited in their ability to project change outside the range seen in recent times (no analog climates).

Experimental studies can provide a mechanistic understanding of how warming will influence patterns of plant diversity and composition. Previous syntheses of these experiments have not sufficiently tested how modulating factors, such as experimental methodology (e.g., spatial scale, duration) and local environmental context, influence plant biodiversity responses. We were able to test for these hypothesized relationships after obtaining a critical mass of primary data from warming experiments around the world, and find support for many of our expectations. We find that climate warming had no overall effect on plant species richness at any spatial scale. However, as expected, warming decreased local plant species richness when the manipulation of temperature was more extreme and when the experiment was conducted under warmer background (summer) temperature conditions. Furthermore, the scale‐dependent effects of warming on species evenness were only seen in studies conducted across longer durations. Experimental warming significantly affected species composition, mostly through turnover of species between warmed and control treatments. These findings offer crucial insights into which ecosystems will respond most dramatically to climate warming and highlight that responses will be lagged.

Locally, we expected a loss of plant species due to direct effects of temperature increase (i.e., temperatures are outside their climate niche, Román‐Palacios & Wiens, 2020) and/or due to indirect effects of temperature increase (i.e., through competition with warm‐adapted species, Eskelinen et al., 2026), with these effects intensifying as the magnitude of the experimental warming increases. Our results that local loss of plant species (i.e., stronger declines in species richness) becomes greater with stronger experimental temperature increase were consistent with this expectation and are in line with a recent meta‐analysis of plant diversity responses to CO_2_, climate change (including waring), and nitrogen addition (Zheng et al., 2026). Previous research has shown that many warming experiments impose temperature increases that exceed projections from global circulation models for their locations (Korell et al., 2020). Consequently, our results may be cautiously interpreted as suggesting that future local‐scale decline in species richness under more likely warming scenarios (i.e., 2–4°C by 2100, IPCC, 2022) could be less severe than indicated by studies with the most extreme experimental treatments (around 5°C).

We expected that local plant communities already exposed to warmer background temperatures during the growing season (e.g., semiarid, desert grasslands) would experience stronger declines in species richness, compared with communities exposed to lower background growing season temperature (e.g., tundra). Our results also supported this expectation. Plant communities with warmer background temperature conditions are also often more water limited. Rising temperatures can exacerbate water scarcity passing a threshold that resident plant species cannot tolerate (Román‐Palacios & Wiens, 2020). For example, in our synthesis, high responses to experimental warming were seen in the Chihuahuan Desert of New Mexico and in dry prairie plant communities of Wyoming, both in North America (Collins et al., 2017; Mueller et al., 2016). Stronger local declines in species richness with increases in (soil) temperature and decreases in soil moisture were also found in a recent meta‐analysis by Zheng et al., 2026. While we considered *temperature* manipulation in this synthesis, another synthesis of *precipitation* manipulation experiments showed a similar pattern of stronger biodiversity declines in response to precipitation reduction in drier, water‐limited plant communities (Korell et al., 2021). Together, these results suggest that plant community types exposed to drier and warmer background climate conditions (e.g., dry prairie, dry grasslands) are at high risk of being severely altered by multiple drivers of climate change.

We did not find evidence that warming influences species richness at larger spatial grains (i.e., the site scale), despite a priori expectations that effects could be either positive or negative. A negative effect of warming on species richness at the site scale would be expected if warming homogenizes plant communities across local plots, whereas a positive effect would be expected if different species colonize across warming plots. Similar to our synthesis, the meta‐analysis by Bastazini et al. (2021) found no evidence that warming changed species richness at larger spatial scales. Together, these results suggest that negative effects of warming on biodiversity (including species richness and evenness) are strongest at local scales, and that responses of replicate warming plots can be idiosyncratic, and therefore difficult to predict.

We expected that warming would create more uneven communities, as detected by another meta‐analysis by Gruner et al. (2017). We found that the effect of warming on species evenness is scale and duration dependent. At local (alpha) scales, warming makes communities more uneven as expected, and this result is especially apparent in long‐running experiments (i.e., >10 years duration). This indicates that at the plot level, single or a few species (e.g., warm‐adapted species) are becoming more dominant over time (e.g., Elmendorf et al., 2015; Feeley et al., 2020). This result has important implications for forecasting responses of plant biodiversity and ecosystem functions to global change, as the identity of the dominant plant species in the community and the degree of dominance of this species will influence coexistence of other species in the community and the ecosystem services provided by the community (Avolio et al., 2019; Eskelinen et al., 2026; Zarnetske et al., 2012). At the beta scale, warming experiments that were longer in duration increased evenness, suggesting that warmed plots diverge in the shape of their species abundance distributions (for example, in how strongly they are dominated), indicating higher heterogeneity among patches (Hillebrand et al., 2008). As a consequence, there is little net effect of warming on evenness at the gamma scale (see also Bastazini et al., 2021).

As expected, we found that warming led to changes in species composition in our synthesis. Specifically, warming altered species composition primarily through changes in species turnover rather than in the nestedness component. This result is in accordance with the meta‐analysis by Bastazini et al. (2021) and studies that have documented directional changes in local plant communities related to the success of warm‐adapted (thermophilic) species with rising temperatures (Bergamin et al., 2024; Elmendorf et al., 2015; Feeley et al., 2020). Compositional change likely reflects varying physiological tolerances among species and potential immigration of (regionally) novel species into local plant communities with climate warming (Catford et al., 2020; Haeuser et al., 2017), if dispersal limitation is not present (Harrison, 2020).

We urge caution that while our synthesis is a global one, the studies come primarily from North America and Europe, and studies with the warmest background temperature were desert or semiarid grasslands, whereas studies from subtropical and tropical regions are missing from the dataset. Tropical and subtropical regions are underrepresented in experimental global change research (Song et al., 2019), even though such regions may be strongly affected for example by rising temperatures (Sheldon, 2019). Another limitation of our study is the low predictability of our results. While we found significant results in the directions hypothesized, the changes are subtle (i.e., effect size is small) and there is still much unexplained variation in the dataset. This variation might be due to differences across studies in other aspects of their experimental design, or ecological context (e.g., biotic interactions, proportion of non‐native species) that were not included in our analysis but could help explain plant community responses to changing environmental conditions (see e.g., Eskelinen et al., 2026; Welshofer et al., 2018).

To conclude, our comprehensive synthesis of climate change experiments provides a more complete view on how climate warming influences biodiversity of terrestrial plant communities across multiple scales and with explicit consideration of context‐dependent factors. Our study highlights that communities will not respond uniformly to climate warming, and that responses will be more dramatic in plant communities at already warmer locations. We demonstrate the importance of experimental design decisions, such as the magnitude of the temperature manipulation, and urge ecologists to consider manipulations at levels that are aligned with projections from global circulation models for their locations (see also Korell et al., 2020). Finally, we highlight that effects of warming on plant biodiversity will change with the temporal (the duration of the experiment) and spatial (alpha, beta, gamma) scales of the analysis, and thus investing in experiments in the long term is very important.

## AUTHOR CONTRIBUTIONS

Lotte Korell, Jonathan M. Chase, W. Stanley Harpole, and Tiffany M. Knight together developed the concept and design of the study. Lotte Korell performed the literature review, data analysis, and initial drafting with major inputs from Tiffany M. Knight. Martin Andrzejak helped with the data analysis. Jonathan M. Chase and W. Stanley Harpole provided substantive feedback and ideas to text and figures and all authors substantially revised the paper.

## CONFLICT OF INTEREST STATEMENT

The authors declare no conflicts of interest.

## Supporting information


Appendix S1.

## Data Availability

Data (Korell et al., 2026) are available in Figshare at https://doi.org/10.6084/m9.figshare.32125000.v5. Code (Korell, 2026) is available in Zenodo at https://doi.org/10.5281/zenodo.20825302.
