# Supplementary material for: Synthesis of scale‐dependent plant biodiversity change in climate warming experiments
Source: Ecology. 2026 Sep 22;107(9):e70486. doi: 10.1002/ecy.70486 (PMC13597150; doi:10.1002/ecy.70486)
Supplement: Supplementary file 1 [file ECY-107-e70486-s001.pdf]

Appendix S1

Synthesis of scale-dependent plant biodiversity change in climate warming experiments

Lotte Korell, Martin Andrzejak, Jonathan M. Chase, W. Stanley Harpole
& Tiffany M. Knight

Ecology

Figure S1

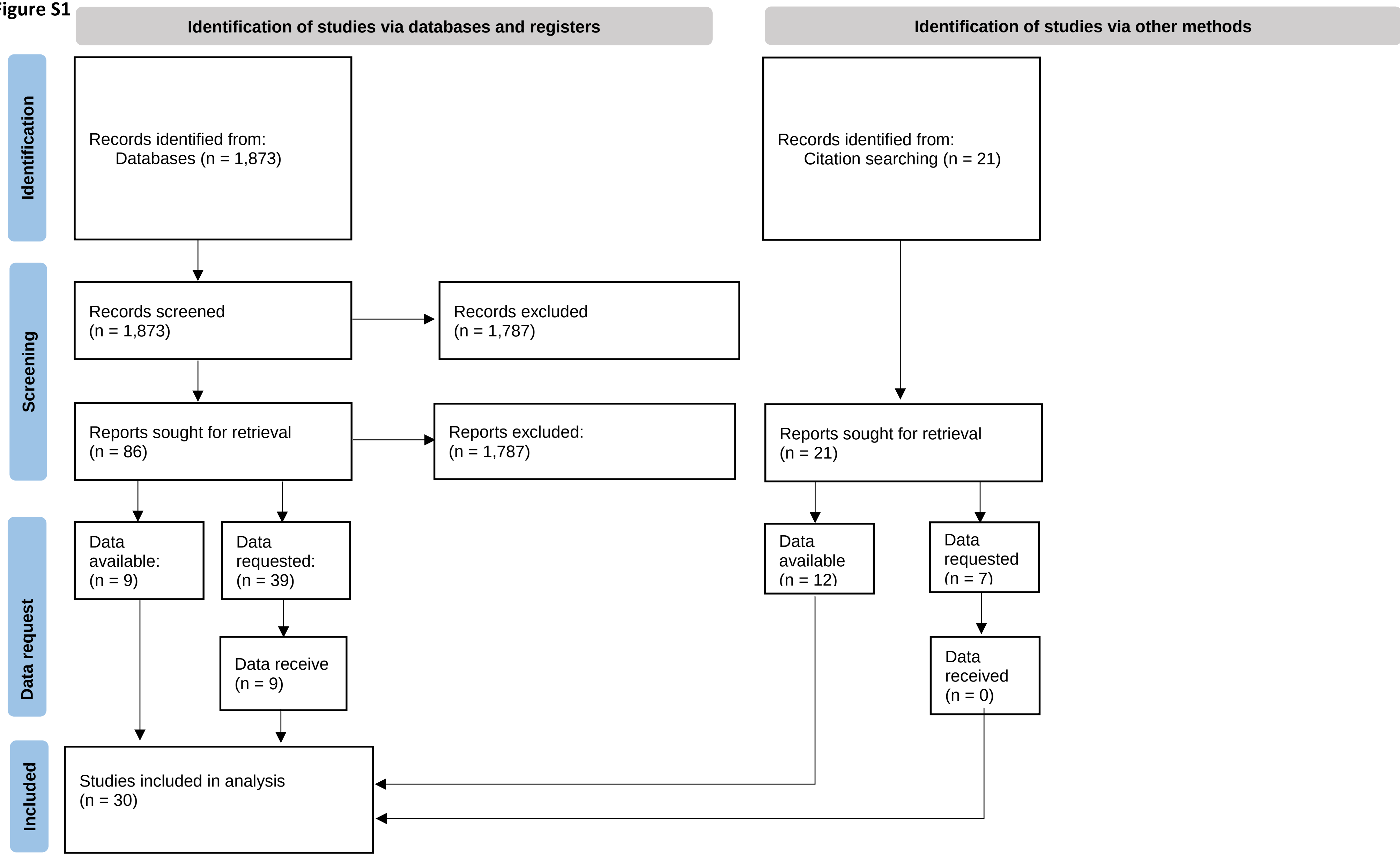

Source: Page, M. J., McKenzie, J. E., Bossuyt, P. M., Boutron, I., Hoffmann, T. C., Mulrow, C. D. et al. The PRISMA 2020 statement: an updated guideline for reporting systematic reviews. *BMJ* 2021; 372: n71. https://doi.org/10.1136/bmj.n71.

Figure S1: Modified PRISMA diagram. Sequence of information in the different phases of the systematic literature search on terrestrial warming experiments following the PRISMA protocol and guidelines.

Table S1: Details about experiments included in the analyses. Study indicates the ID of study, experiment indicates the ID of experimental manipulation included in the analysis whereas type of community/ manipulation describes the community type or manipulation type of the experimental manipulation where each unique combination of site, community type, and Δ T is considered one experiment. Lat and long indicate the latitude and longitude of the site, respectively. Community type considers the main vegetation type the experiment was conducted at. Mean annual precipitation (MAP, mm a-1), mean annual temperature (MAT, ° C), temperature of the warmest quarter (T warm; ° C) and potential evapotranspiration (PET, kg a^{-1}) indicated the climatic conditions of the experimental sites. Magnitude of temperature manipulation (delta T, °C), local-scale plot size (size.local, in square meters) and number of experimental units (replicates) and the duration of the experimental treatment (in years) are given. Reference indicates the citation to the study as listed in the `References´ on the next page of this document.

#Study	#Exp	#site	latitude	longitude	Community type	MAT	MAP	T warm	delta T	size.local	replicates	duration	Study name	Reference
1	1	1	74.47	-20.55	tundra (dry)	-12.02	179	2.55	1.5	1	5	4	ITEX_10	1
1	2	1	74.47	-20.55	tundra (mesic)	-12.02	179	2.55	1.5	1	5	4	ITEX_10	1
2	4	2	-42.34	147.34	grassland (montane)	12.59	680	14.25	3.33	1	8	1	Britton_2021	2
2	3	3	-42.09	147.09	grassland (sub-alpine)	9.37	713	11.75	3.33	1	8	1	Britton_2021	2
3	5	4	53.19	18.31	peatland	8.21	542	17.25	1.27	0.16	3	2	Buttler_2023	3
4	6	5	68.62	-149.61	tundra (arctic)	-12.26	249	8.65	5	0.16	8	10	Arctic_LTER	4
5	7	6	34.33	-106.72	grassland (desert)	17.55	291	24.85	1.1	1	5	10	Collins_2017	5
6	8	7	40.05	-105.58	tundra (alpine)	-1.72	501	10.05	1	1	6	16	Collins_2022	6
7	9	8	78.10	15.48	tundra (mesic)	-8.64	341	3.35	1.5	0.5625	5	3	ITEX_4	1
8	10	8	78.10	15.48	tundra (wet)	-8.64	341	3.35	1.5	0.5625	5	3	ITEX_4	1
9	11	9	40.05	-105.58	tundra (alpine)	-1.72	501	10.05	1	1	6	10	Farrer_2015	7
10	12	10	62.07	-6.96	grassland (alpine)	5.02	2327	7.85	0.771	0.25	10	6	ITEX_2	1
11	13	11	68.63	-149.64	tundra (wet)	-12.13	243	8.75	5	1	6	3	Arctic_LTER_1	8
12	14	12	53.23	-1.90	grassland (limestone)	9.51	1001	13.55	3	2.5	5	16	Grime_2008	9
13	15	13	46.47	9.58	tundra (apine)	-2.29	1314	5.85	0.93	0.8	9	2	ITEX_6	1
14	16	14	79.16	-75.26	tundra (arctic)	-19.2	109	1.95	1.3	1	10	3	ITEX_11	1
15	18	15	62.18	9.37	tundra	0.05	438	9.95	1.5	0.25	10	10	ITEX_1	1
16	17	16	70.45	-157.41	tundra (dry)	-13.33	138	7.65	1.46	0.75	24	22	Hollister_2015	10
16	19	17	71.32	-156.60	tundra (dry)	-13.1	117	3.25	1.46	0.75	24	24	Hollister_2015	10
16	20	16	70.45	-157.40	tundra (wet)	-13.33	138	7.65	1.46	0.75	24	22	Hollister_2015	10
16	21	17	71.32	-156.60	tundra (wet)	-13.1	117	3.25	1.46	0.75	24	23	Hollister_2015	10
17	22	18	68.21	18.49	tundra	-4.33	671	7.05	0.55	0.25	5	8	ITEX_9	1
18	23	20	61.20	-138.30	tundra	-8.44	393	5.55	1.5	0.25	8	10	ITEX_3	1
19	24	21	42.83	-104.90	grassland	9.47	419	20.15	1.5	1.5	5	7	Mueller_2016	11
20	25	22	60.62	7.52	tundra	-2.04	1071	7.35	1.5	0.6	5	12	Olsen_2014	12
21	26	23	42.28	-123.64	grassland (prairie)	14.16	1261	20.95	2.5	1	5	3	Reed_2021	13
21	27	24	44.03	-123.18	grassland (prairie)	14.73	1056	18.45	2.5	1	5	3	Reed_2021	13
21	28	25	46.90	-122.74	grassland (prairie)	13.6	1225	16.95	2.5	1	5	3	Reed_2021	13
22	29	26	68.21	18.49	tundra (wet)	-4.33	671	7.05	3.9	1.25	5	10	ITEX_8	1
23	30	27	69.10	20.80	tundra	-1.46	377	10.05	1.2	0.25	6	15	ITEX_12	1
24	31	28	51.13	100.92	grassland (steppe)	-7.44	451	8.85	1.5	0.5	8	4	Ronk_2019	14
24	32	28	51.13	100.92	grassland (steppe)	-7.44	451	8.85	1.5	0.5	7	4	Ronk_2019	14
25	33	29	34.98	97.52	grassland	-4.48	328	6.95	4	1	6	13	Shi_2015	15
26	34	30	68.21	18.49	tundra (dry)	-4.33	671	7.05	3.9	0.25	6	20	ITEX_7	1
27	35	31	29.91	102.01	grassland (high alpine)	2.09	959	7.85	1.5	0.25	7	4	Vandvik_2020	16
27	36	32	29.89	102.02	grassland (alpine)	3.69	895	9.15	1.5	0.25	7	4	Vandvik_2020	16
27	37	33	29.86	102.04	grassland (middle)	5.09	829	10.25	1.5	0.25	7	4	Vandvik_2020	16
27	38	34	29.84	102.03	grassland (lowland)	7.22	718	11.85	1.5	0.25	7	4	Vandvik_2020	16
28	39	35	70.82	147.48	tundra	-16.01	211	7.75	0.7	0.25	5	3	Wang_2017	17
29	40	36	42.40	-85.40	grassland	11.04	884	20.95	1.73	1	3	2	Welshofer_2018	18
29	41	37	45.56	-84.71	grassland	7.32	811	18.85	1.84	1	3	2	Welshofer_2018	18
30	42	38	42.03	116.28	grassland (steppe)	3.94	372	18.75	0.98	1	6	4	Yang_2011	19

References

1. Elmendorf, S. C., G. H. R. Henry, R. D. Hollister, R. G. Björk, A. D. Bjorkman, T. V. Callaghan, L. S. Collier, E. J. Cooper, J. H. C. Cornelissen, T. A. Day, A. M. Fosaa, W. A. Gould, J. Grétarsdóttir, J. Harte, L. Hermanutz, D. S. Hik, A. Hofgaard, F. Jarrad, I. S. Jónsdóttir, F. Keuper, K. Klanderud, J. A. Klein, S. Koh, G. Kudo, S. I. Lang, V. Loewen, J. L. May, J. Mercado, A. Michelsen, U. Molau, I. H. Myers-Smith, S. F. Oberbauer, S. Pieper, E. Post, C. Rixen, C. H. Robinson, N. M. Schmidt, G. R. Shaver, A. Stenström, A. Tolvanen, Ø. Totland, T. Troxler, C.-H. Wahren, P. J. Webber, J. M. Welker, and P. A. Wookey. 2012. Global Tundra Vegetation Change - 30 years of plant abundance data from unmanipulated and experimentally-warmed plots (access date: 20.08.2018). Canadian Cryospheric Information Network., www.polardata.ca.
2. Britton, T. G., M. J. Hovenden, M. Porter, A. Flittner, R. Brinkhoff, and M. M. Mayfield. 2021. Plant communities, populations and individuals have distinct responses to short-term warming and neighbour biomass removal in two montane grasslands. Applied Vegetation Science **24**:e12557.
3. Buttler, A., L. Bragazza, F. Laggoun-Défarge, S. Gogo, M.-L. Toussaint, M. Lamentowicz, B. H. Chojnicki, M. Słowiński, S. Słowińska, M. Zielińska, M. Reczuga, J. Barabach, K. Marcisz, Ł. Lamentowicz, K. Harenda, E. Lapshina, D. Gilbert, R. Schlaepfer, and V. E. J. Jassey. 2023. Ericoid shrub encroachment shifts aboveground–belowground linkages in three peatlands across Europe and Western Siberia. Global Change Biology **29**:6772-6793.
4. Shaver, G. R., and F. S. Chapin III. 1991. Production: Biomass Relationships and Element Cycling in Contrasting Arctic Vegetation Types. Ecological Monographs **61**:1-31
5. Collins, S. L., L. M. Ladwig, M. D. Petrie, S. K. Jones, J. M. Mulhouse, J. R. Thibault, and W. T. Pockman. 2017. Press–pulse interactions: effects of warming, N deposition, altered winter precipitation, and fire on desert grassland community structure and dynamics. Global Change Biology **23**:1095-1108.
6. Collins, C. G., S. C. Elmendorf, J. G. Smith, L. Shoemaker, M. Szojka, M. Swift, and K. N. Suding. 2022. Global change re-structures alpine plant communities through interacting abiotic and biotic effects. Ecology letters **25**:1813-1826.
7. Farrer, E. C., I. W. Ashton, M. J. Spasojevic, S. Fu, D. J. X. Gonzalez, and K. N. Suding. 2015. Indirect effects of global change accumulate to alter plant diversity but not ecosystem function in alpine tundra. Journal of Ecology **103**:351-360.
8. Gough, L., and S. E. Hobbie. 2003. Responses of moist non-acidic arctic tundra to altered environment: productivity, biomass, and species richness. Oikos **103**:204-216.
9. Grime, J. P., J. D. Fridley, A. P. Askew, K. Thompson, J. G. Hodgson, and C. R. Bennett. 2008. Long-term resistance to simulated climate change in an infertile grassland. Proceedings of the National Academy of Sciences **105**:10028-10032.
10. Hollister, R. D., P. J. Webber, and C. E. Tweedie. 2005. The response of Alaskan arctic tundra to experimental warming: differences between short- and long-term responses. Global Change Biology **11**:525-536.
11. Mueller, K. E., D. M. Blumenthal, E. Pendall, Y. Carrillo, F. A. Dijkstra, D. G. Williams, R. F. Follett, and J. A. Morgan. 2016. Impacts of warming and elevated CO 2 on a semi-arid grassland are non-additive, shift with precipitation, and reverse over time. Ecology letters **19**:956-966.
12. Olsen, S. L., and K. Klanderud. 2014. Exclusion of herbivores slows down recovery after experimental warming and nutrient addition in an alpine plant community. Journal of Ecology **102**:1129-1137.
13. Reed, P. B., L. E. Pfeifer-Meister, B. A. Roy, B. R. Johnson, G. T. Bailes, A. A. Nelson, and S. D. Bridgham. 2021. Introduced annuals mediate climate-driven community change in Mediterranean prairies of the Pacific Northwest, USA. Diversity and Distributions **27**:2584-2595.
14. Ronk, A., P. Liancourt, B. Boldgiv, P. S. Petraitis, and B. B. Casper. 2020. Greater effect of warming on community composition with increased precipitation and in moister landscape location. Journal of Vegetation Science **31**:3-13.
15. Shi, Z., R. Sherry, X. Xu, O. Hararuk, L. Souza, L. Jiang, J. Xia, J. Liang, and Y. Luo. 2015. Evidence for long-term shift in plant community composition under decadal experimental warming. Journal of Ecology **103**:1131-1140.
16. Vandvik, V., A. H. Halbritter, Y. Yang, H. He, L. Zhang, A. B. Brummer, K. Klanderud, B. S. Maitner, S. T. Michaletz, and X. Sun. 2020. Plant traits and vegetation data from climate warming experiments along an 1100 m elevation gradient in Gongga Mountains, China. Scientific Data **7**:189.
17. Wang, P., J. Limpens, L. Mommer, J. van Ruijven, A.L. Nauta, F. Berendse, G. Schaepman-Strub, D. Blok, T.C. Maximov, and M.M.P.D. Heijmans. 2017. Above- and below-ground responses of four tundra plant functional types to deep soil heating and surface soil fertilization. Journal of Ecology **105**: 947-957.
18. Welshofer, K. B., P. L. Zarnetske, N. K. Lany, and Q. D. Read. 2018. Short-term responses to warming vary between native vs. exotic species and with latitude in an early successional plant community. Oecologia **187**:333-342.
19. Yang, H., M. Wu, W. Liu, Z. Zhang, N. Zhang, and S. Wan. 2011. Community structure and composition in response to climate change in a temperate steppe. Global Change Biology **17**:452-465.

Table S2: Summary statistics for the highest-ranked mixed effects model (delta AICc < 2) for the response variables species richness, effective number of species (ENS PIE) across scales (alpha, beta, gamma). Degrees of freedom (df), log likelihood (logLik), delta AICc, AICc weights and conditional variance explained (cR2) and marginal variance explained (mR2) are shown. Models include following variables: magnitude of temperature manipulation (delta T, °C), study duration (years), and temperature of the warmest quarter (T warm, °C). '+' indicates an additive effect.

Response	scale	moderators	df	logLik	AICc	delta	weight	cR^2	mR^2
Species richness	alpha	delta T + duration + T warm	6	1.47	9.300	0.000	0.406	0.200	0.062
	beta	Null	3	17.76	-29.44	0.000	0.420	0.041	-
		T warm	4	18.25	-28.37	1.070	0.240	0.057	0.004
		delta T	4	17.95	-27.79	1.65	0.18	0.042	0.001
		duration	4	17.8	-27.47	1.97	0.16	0.058	0.001
	gamma	delta T	4	16.08	-23.08	0.000	0.210	0.212	0.083
		delta T + duration	5	17.37	-23.07	0.010	0.210	0.307	0.149
		duration	4	15.72	-22.37	0.720	0.150	0.268	0.085
		delta T + duration + T warm	6	18.25	-22.11	0.980	0.130	0.326	0.186
		Null	3	14.36	-22.10	0.990	0.130	0.100	-
		duration + T warm	5	16.60	-21.53	1.560	0.100	0.264	0.118
		delta T + T warm	5	16.46	-21.25	1.840	0.080	0.263	0.124
Evenness (ENS Pie)	alpha	duration	4	-185.36	378.84	0.000	0.530	0.034	0.016
		delta T + duration	5	-185.05	380.27	1.430	0.260	0.038	0.017
		delta T + T warm	5	-185.26	380.71	1.860	0.210	0.040	0.016
	beta	duration + T warm	5	-182.73	375.64	0.000	0.370	0.049	0.028
		delta T + duration + T warm	6	-181.93	376.10	0.460	0.290	0.049	0.031
		duration	4	-184.47	377.05	1.410	0.180	0.038	0.017
		delta T	5	-183.55	377.27	1.630	0.160	0.053	0.004
	gamma	T warm	4	32.91	-56.74	0.000	0.550	0.107	0.107
		delta T + T warm	5	33.36	-55.05	1.700	0.240	0.122	0.122
		duration + T warm	5	33.27	-54.87	1.870	0.220	0.136	0.117
